# The complete mitogenome of an undescribed clam shrimp of the genus *Gondwanalimnadia* (Branchiopoda: Spinicaudata), from a temporary wetland in Central District, Botswana

**DOI:** 10.1080/23802359.2020.1731351

**Published:** 2020-02-28

**Authors:** Murphy Tladi, Tatenda Dalu, D. Christopher Rogers, Casper Nyamukondiwa, Arsalan Emami-Khoyi, Jody C. Oliver, Peter R. Teske, Ryan J. Wasserman

**Affiliations:** a Department of Biological Sciences and Biotechnology, Botswana International University of Science and Technology, Palapye, Botswana;; b Department of Ecology and Resource Management, University of Venda, Thohoyandou, South Africa;; c Kansas Biological Survey and the Natural History Museum (Biodiversity Institute), Lawrence, KS, USA;; d Centre for Ecological Genomics and Wildlife Conservation, Department of Zoology, University of Johannesburg, Auckland Park, South Africa

**Keywords:** African Spinicaudata, Illumina, mitochondrial DNA, phylogenetics, Crustacea

## Abstract

Clam shrimps (Spinicaudata) are a widespread and diverse crustacean group that frequent temporary aquatic habitats, but few complete mitochondrial genomes have been published for this group. Here, we report the mitogenome of an undescribed *Gondwanalimnadia* species from Botswana. Raw sequences were assembled into a single circular genome with a total length of 15,663 bp. Thirteen protein-coding genes, 22 tRNAs, and 2 rRNAs were identified using the MITOS pipeline. The mitogenome’s GC content is 33.52%. Phylogenetic analysis using protein-coding genes confirmed that *Gondwanalimnadia* sp. is closely related to another member of the Limnadiidae, *Limnadia lenticularis*.

Spinicaudatans are an order of freshwater crustaceans commonly known as spiny clam shrimps. These large branchiopod crustaceans occur exclusively in seasonally astatic aquatic habitats (Brendonck et al. 2008). Similar to other branchiopods, clam shrimp rapidly attain sexual maturity and within short hydroperiod windows, produce dormant eggs that can withstand dry periods (Brendonck et al. 2008; Rogers 2009). They are thought to be omnivores with diets consisting of detritus, plankton and algae (Hethke et al. 2019). *Gondwanalimnadia* spp. belong to the Limnadiidae (Weeks et al. 2012; Rogers et al. 2016), a family in the order Spinicaudata with a global distribution, except for Antarctica (Bellec and Rabet 2016). As with many spinicaudatan families, little is known about their genomic make-up, and their correct placement within the branchiopod phylogeny is not fully resolved (Bellec and Rabet 2016). Here, we describe the complete mitogenome of an undescribed *Gondwanalimnadia* species obtained from Botswana, and investigate its phylogenetic placement among several other brachiopods.

Specimens of the undescribed *Gondwanalimnadia* species were collected from a temporary pond on the outskirts of Palapye, Central district, Botswana (GPS 27.16616 E, 22.54507 S) and preserved in 80% ethanol. Voucher specimens from the locality were deposited at the Kansas Biological Survey (DCR-1138). Genomic DNA of high molecular weight was extracted using the CTAB method (Doyle and Doyle 1987). An indexed DNA library was constructed using NEBNext DNA Library Preparation Kit (Massachusetts, USA), and 1 µg of genomic DNA as template. The DNA library was sequenced on the Illumina Hi-Seq platform (California, USA) using 2 × 150 chemistry with an average insert size of 350 bp.

 The Illumina sequencing run yielded a total of 23,096,464 raw sequences. The complete mitogenome was assembled using NOVOPlasty v3.5 (Dierckxsens et al. 2016) and annotated using MITOS Web Server (Bernt et al. 2013). The assembly resulted in a single circular genome with a total length of 15,663 bp. The MITOS pipeline identified 13 protein-coding genes, 22 tRNAs, and 2 rRNAs, typical of all crustaceans. The GC content of the assembly was estimated at 33.52%. Several instances of non-canonical start codon and incomplete stop codons were observed, as for other arthropods (Monsanto et al. 2019; Jagatap et al. 2019). Protein coding sequences from the studied species, eight closely related species with complete mitogenomes in the NCBI database, and an outgroup (*Dysdercus evanescens*) were aligned in MAFFT v7.429 (Katoh et al. 2009). A Bayesian phylogenetic tree was reconstructed using package BEAST2 (Bouckaert et al. 2014). The program’s default settings were used, except that the substitution model was set to HKY (Hasegawa et al. 1985) with four gamma categories. BEAST2 was run for 50,000,000 iterations with 30% burn-in. Markov chain convergence and Effective Sample Size (ESS) were visually inspected in Tracer v1.7 (Rambaut et al. 2018). The phylogenetic tree was visualized in FigTree v1.4 (Rambaut and Drummond 2012), placing the uncharacterized *Gondwanalimnadia* sp., along with another limnadiid (*Limnadia lenticularis*), in a monophyletic clade with cladoceran and notostracan representatives (Figure 1).

**Figure 1.**
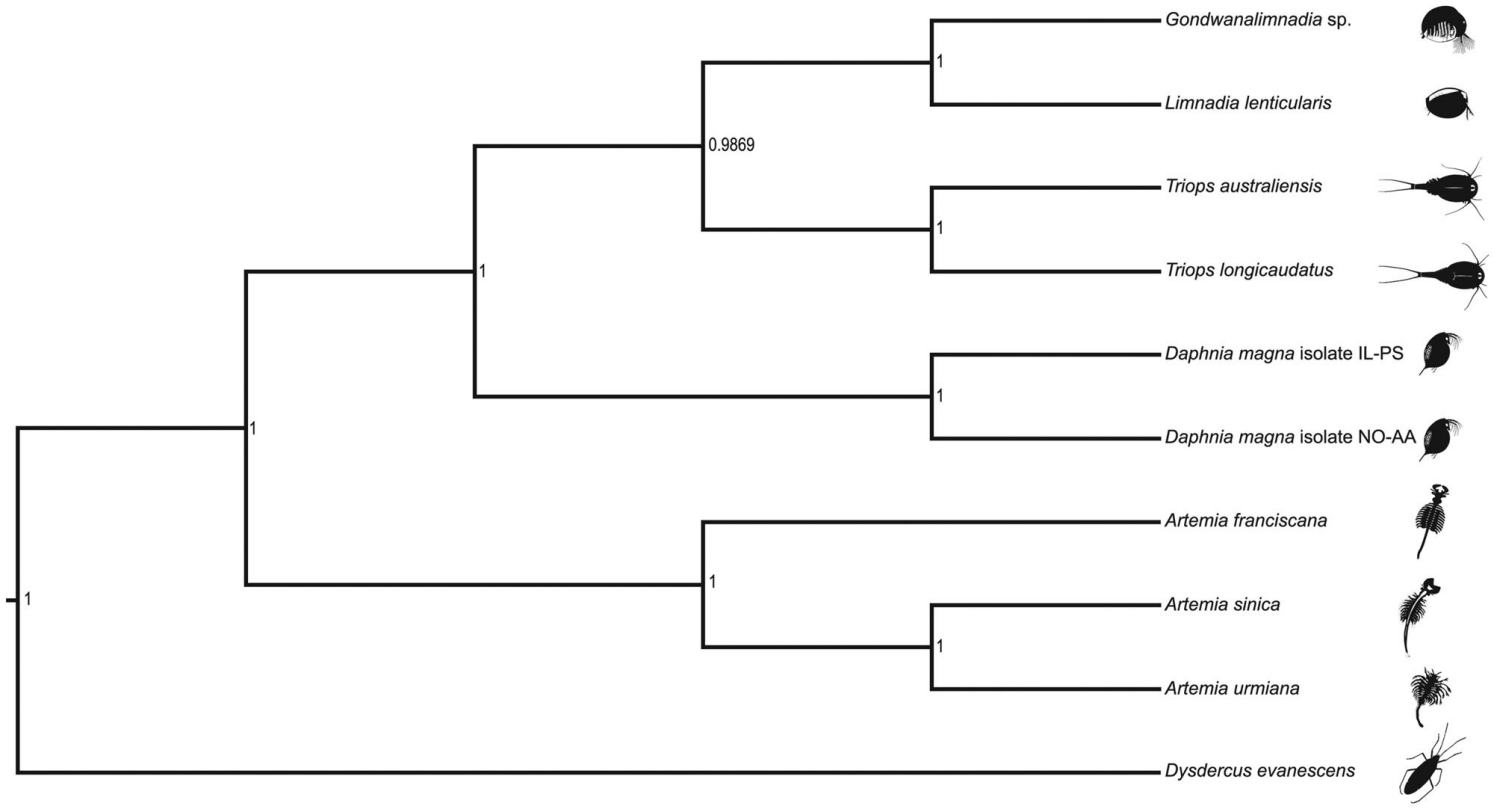
A Bayesian phylogenetic tree constructed in BEAST2 using mitogenome sequences of an undescribed *Gondwanalimnadia* species (NCBI accession number MN625703) and nine related species. *Daphnia magna* isolate IL-PS: MH683649.1*, Daphnia magna* isolate NO-AA: MH683655.1, *Limnadia lenticularis*: NC_039394.1, *Triops australiensis*: LK391946.1, *Triops longicaudatus*: AY639934.1*, Dysdercus evanescens*: NC_042437.1, *Artemia urmiana:* NC_021382.1, *Artemia sinica*: NC_042147.1, *Artemia franciscana*: X69067.1. The numbers on the tree indicate the posterior probability estimated for each node.
